# An Unusual Complication in Osteonecrosis of Femoral Head: A Case Report

**DOI:** 10.1155/2013/313289

**Published:** 2013-01-17

**Authors:** Singaravadivelu Vaidyanathan, Yuvaraja Murugan, Kingsly Paulraj

**Affiliations:** Institute of Orthopedics and Traumatology, Madras Medical College, Chennai 600003, India

## Abstract

We report a case of fracture of femoral head occurring in the setting of underlying osteonecrosis following a low-energy trauma in a middle-aged male. Patient also has underlying pelvic pathology from a previous trauma. The possible mechanism of injury is discussed. Extensive literature search reveals that this is the first report of a fracture in an osteonecrotic femoral head.

## 1. Introduction

Osteonecrosis of head of femur may be idiopathic or result from several causes such as alcohol abuse, chronic steroid use, trauma, renal osteodystrophy, hypercoagulable states, gout, and so forth [1]. Fracture of head of femur is usually a high-energy injury associated with dislocation of the hip joint [2]. We report a case of femoral head fracture following a low energy trauma in a middle aged male with preexisting hip and pelvic pathology. Fracture occurring in an osteonecrotic head of femur is a complication unreported so far, to the best of our knowledge.

## 2. Case History

A 55-year-old male presented to us with history of pain right hip and limp for the past 12 years, with inability to walk for the past 10 days following a low energy trauma.

 The patient had past history of road traffic accident 15 years ago when he sustained injury to his right hemipelvis and was treated with upper tibial pin traction for 6 weeks. He was ambulant and pain free later. Past medical records were not available.

 He developed dull ache in his right hip and limp 3 years later. Pain was deep seated, aggravated on activities, and partially relieved with analgesics. The patient was able to walk unaided and perform activities of daily living and squat in Indian toilet.

 He sustained a low energy trauma while riding bicycle 10 days back, falling on his right hip. He experienced sharp pain with inability to weight bear on his right leg with restriction of right hip movements.

 The patient is a chronic alcoholic, taking about 300 mL/week over the past 30 years. CAGE questionnaire [3] revealed 3 positive responses out of 4, indicating significant alcohol dependence. He is a nonsmoker. He has no history of hypertension, diabetes, tuberculosis, asthma, or chronic drug intake.

 Examination revealed a moderately built patient with stable vital parameters. Right side ASIS was elevated. Anterior joint tenderness and crepitus were present in right hip. He had fixed flexion deformity of 20 degrees, and all other movements of right hip were painful and restricted. 1 cm of supratrochanteric shortening was present.

 Investigations revealed normal blood parameters. 

**Figure 1:**
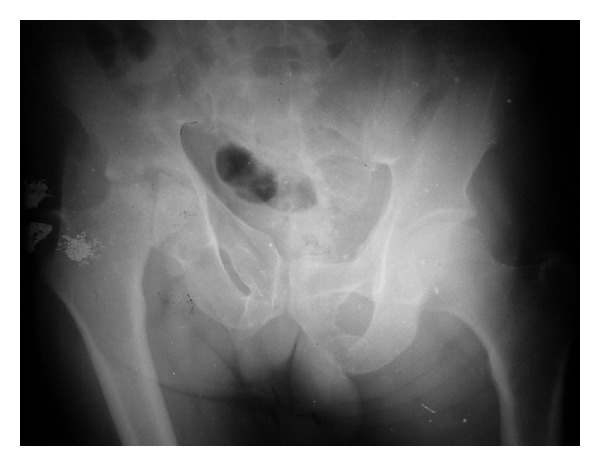
X-ray pelvis with both hips showing collapse of right femur head with fracture of head of femur. Evidence of old vertical shear type sacroiliac disruption right side is seen.

 Plain X-ray pelvis with both hips revealed old vertical shear injury to right sacroiliac joint evidenced by cephalic migration of right hemipelvis; this is the result of previous trauma. The right hip showed superior articular surface irregularity with collapse of right femoral head. Fracture head of femur was found extending from superior to inferior articular surface (Figure 1). The left hip was found to be normal.

**Figure 2:**
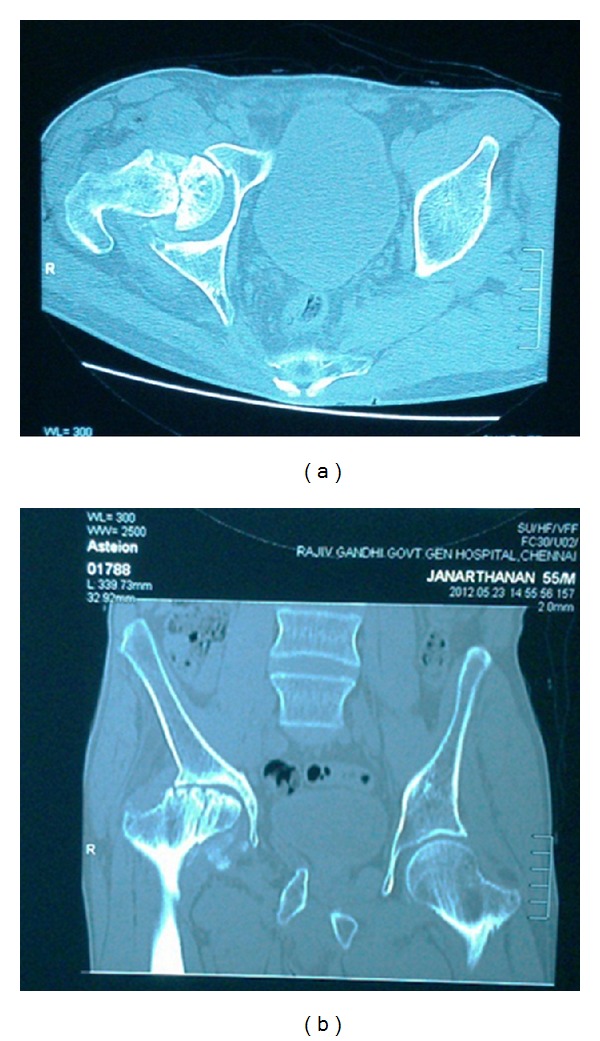
CT images revealing fracture of head of femur.

 CT pelvis with both hips with 3D reconstruction confirmed the fracture line in head of femur (Figure 2).

**Figure 3:**
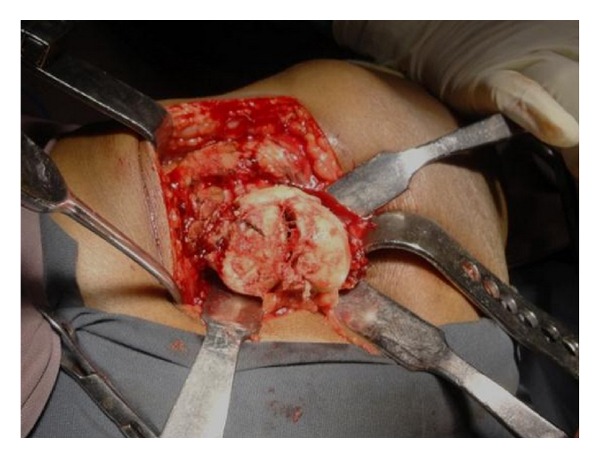
Intraoperative picture shows fractured and deformed femoral head.

**Figure 4:**
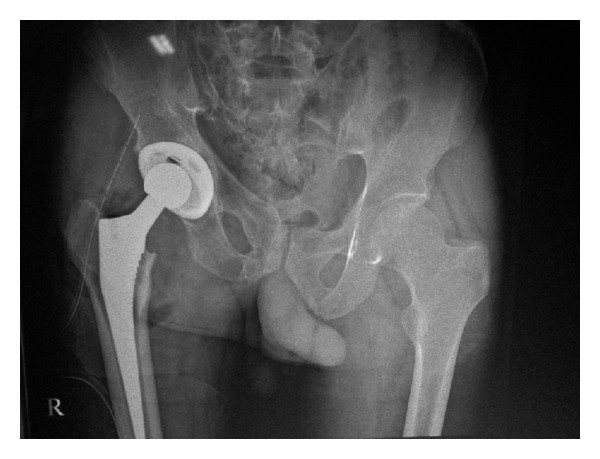
Postoperative radiograph.

A diagnosis of fracture head of femur right hip with underlying osteonecrosis was made based on history and imaging findings. Uncemented total hip replacement was planned due to gross destruction of femur head and acetabular degenerative changes. Intraoperatively, a deformed and fractured femoral head was found and was delivered out of acetabulum and sent for histopathological examination (Figure 3). Reconstruction was done with 52 mm Duraloc shell, polyethylene liner, and 11 size CORAIL femur stem (DePuy Johnson and Johnson) (Figure 4).

**Figure 5:**
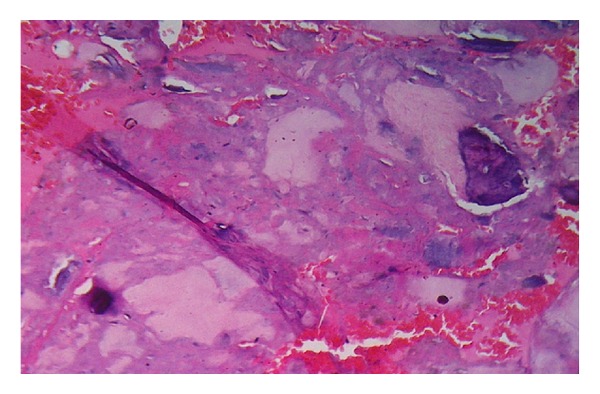
Histopathology slide showing necrotic bone with reparative fibroblastic proliferation.

Histopathology of resected specimen revealed necrotic bone with reparative fibroblastic proliferation—consistent with osteonecrosis (Figure 5).

## 3. Discussion

Initial trauma 15 years ago was a vertical shear injury with right sacroiliac disruption. This is not likely to have caused the present symptoms of the patient.

Osteonecrosis of femoral head is probably due to chronic alcoholism which is a well-established risk factor for osteonecrosis [4, 5]. His chronic symptoms can be attributed to secondary degenerative osteoarthritis following osteonecrosis.

The patient was able to carry out activities of daily living with moderate pain and discomfort. Trivial trauma sustained recently due to fall from bicycle resulted in severe restriction of activities and led him to seek medical attention. Present X-ray revealed fracture of femoral head with underlying osteonecrosis.

Fracture of femur head is usually reported in high-energy injuries associated with hip dislocations, so much so that both Pipkin and Brumback classification schemes for femoral head fractures describe only those associated with hip dislocations [2, 6]. There have been very few reports of low energy injuries causing femoral head fractures without hip dislocation but none of that of a fracture in an avascular head [7–10]. In our patient, fall onto his right side and thereby transmission of force from trochanteric region along femoral neck might have caused failure in head region with resulting fracture. Normally, such mode of injury causes fracture of neck of femur in geriatric population due to osteoporosis. But, Mody and Wainwright (1996) proposed that in a relatively young person without osteoporosis the force is directly transferred to the head region [7].

Fracture head of femur may be treated by either internal fixation or arthroplasty. In our patient arthroplasty was chosen due to underlying hip arthritis. 

 This case is being presented for the unreported, rare complication occurring in a relatively common clinical condition.
